# Spontaneous pneumomediastinum: a surrogate of P-SILI in critically ill COVID-19 patients

**DOI:** 10.1186/s13054-022-04228-1

**Published:** 2022-11-12

**Authors:** Alexandre Elabbadi, Tomas Urbina, Enora Berti, Damien Contou, Gaëtan Plantefève, Quintana Soulier, Audrey Milon, Guillaume Carteaux, Guillaume Voiriot, Muriel Fartoukh, Aude Gibelin

**Affiliations:** 1 Assistance Publique – Hôpitaux de Paris, Service de Médecine Intensive Réanimation, Hôpital Tenon, APHP, Sorbonne Université, 4 Rue de la Chine, 75020 Paris, France; 2 Assistance Publique – Hôpitaux de Paris, Service de Médecine Intensive Réanimation, Hôpital Saint-Antoine, Sorbonne Université, Paris, France; 3 Assistance Publique – Hôpitaux de Paris, DMU Médecine, Service de Médecine Intensive Réanimation, Hôpital Henri Mondor, Hôpitaux Universitaires Henri Mondor, Créteil, France; 4 Service de Réanimation Polyvalente, Centre Hospitalier Victor Dupouy, Argenteuil, France; 5 Assistance Publique – Hôpitaux de Paris, Service de Radiologie, Hôpital Tenon, Sorbonne Université, Paris, France

**Keywords:** Pneumomediastinum, Coronavirus disease 2019, Pneumonia, Intensive care unit

## Abstract

Spontaneous pneumomediastinum (SP) has been described early during the COVID-19 pandemic in large series of patients with severe pneumonia, but most patients were receiving invasive mechanical ventilation (IMV) at the time of SP diagnosis. In this retrospective multicenter observational study, we aimed at describing the prevalence and outcomes of SP during severe COVID-19 with pneumonia before any IMV, to rule out mechanisms induced by IMV in the development of pneumomediastinum.

Among 549 patients, 21 patients (4%) developed a SP while receiving non-invasive respiratory support, after a median of 6 days [4–12] from ICU admission. The proportion of patients requiring IMV was similar. However, the time to tracheal intubation was longer in patients with SP (6 days [5–13] vs. 2 days [1–4]; *P* = 0.00002), with a higher first-line use of non-invasive ventilation (*n* = 11; 52% vs. *n* = 150; 28%; *P* = 0.02). The 21 patients who developed a SP had persisting signs of severe lung disease and respiratory failure with lower ROX index between ICU admission and occurrence of SP (3.94 [3.15–5.55] at admission vs. 3.25 [2.73–4.02] the day preceding SP; *P* = 0.1), which may underline potential indirect signals of Patient-self inflicted lung injury (P-SILI).

In this series of critically ill COVID-19 patients, the prevalence of SP without IMV was not uncommon, affecting 4% of patients. They received more often vasopressors and had a longer ICU length of stay, as compared with their counterparts. One pathophysiological mechanism may potentially be carried out by P-SILI related to a prolonged respiratory failure, as underlined by a delayed use of IMV and the evolution of the ROX index between ICU admission and the day preceding SP.

## Introduction

Spontaneous pneumomediastinum (SP) is defined by the presence of air within the mediastinum without traumatic lesion [1]. It has been described during ARDS even in the era of protective ventilation [2].

Large COVID-19 series have reported SP during severe pneumonia, but most patients were receiving invasive mechanical ventilation (IMV) at the time of SP diagnosis. We aimed at describing the prevalence of SP during severe COVID-19 with pneumonia before any IMV, in order to rule out mechanisms induced by IMV in the development of pneumomediastinum, and at investigating its prognostic impact.

## Methods

We conducted a retrospective multicenter observational study in four French intensive care units (ICUs) between August 2020 and April 2021. All patients with severe COVID-19 with pneumonia defined by laboratory-confirmed SARS-CoV-2 severe pneumonia, the presence of acute respiratory failure (defined as a respiratory rate over 25 breaths/min or other signs of respiratory distress including active abdominal breathing, paradoxical breathing, impaired consciousness) and hypoxemia requiring oxygen therapy or non-invasive ventilation, were included. Spontaneous pneumomediastinum was diagnosed either on chest X-ray or chest CT-scan by an independent radiologist (AM), who analyzed the CT scan lesions according to the European Society of Radiology [3], including the extent of lesions related to SARS-CoV-2 infection and the presence of emphysema or fibrotic lesions that may have favored the development of pneumomediastinum. The study period was selected because of the standardization of the management of patients with severe COVID-19 after the first wave, including high flow nasal oxygen (HFNO) and systemic steroids [4]. The investigators of each center identified eligible patients and collected the data from medical records.

The primary endpoint was to estimate the prevalence of SP during COVID-19 with severe pneumonia in patients with a non-invasive respiratory support. Secondary endpoints were to investigate the possible risk factors associated with SP, as well as its prognosis impact.

The patients who required IMV on ICU admission and those who developed pneumomediastinum during IMV were excluded from the study. Statistical analysis was performed with R (version 4.0.4 (2021-02-15).

## Results

During the study period, 672 patients with COVID-19 with severe pneumonia were screened. A total of 123 patients were excluded from analysis: 110 patients required IMV on ICU admission, 11 patients developed a pneumomediastinum after intubation, and 2 patients developed a pneumothorax while receiving IMV. No patient had isolated pneumothorax (without SP) before intubation, except one patient with a necrotizing and bacteremic pneumococcal pneumonia associated with pleural empyema.

Finally, 549 patients were included. They were 377 (69%) males with a median age of 64 [56–71] years, with frequent comorbidities mainly arterial hypertension (*n* = 308; 56%) and diabetes (*n* = 190; 35%), and moderate overweight (median body-mass index 28.7 [25–32.7] kg/m^2^). Baseline characteristics are shown in Table 1. Twenty-one patients (4%) developed a SP while under non-invasive respiratory support, with a median of 9 days [7–23] after COVID-19 symptoms onset, and of 6 days [4–12] from ICU admission. A pneumothorax was associated with SP in six patients (29%), three of whom required a chest tube drainage. Among those 21 patients with SP under non-invasive respiratory support, 11 (52%) were intubated after 1 day [0–3] of SP diagnosis.

**Table 1:** Baseline characteristics, treatments and outcomes of ICU patients with severe COVID-19 pneumonia

	All patients (*n* = 549)	Spontaneous pneumomediastinum (*n* = 21)	No Spontaneous pneumomediastinum (*n* = 528)	*P* Value
Age (year)	64 [56–71]	62[54–70]	64[56–71]	0.34
Sex male	377 (68.7)	15 (71.4)	362 (68.6)	0.78
Current smoking	23 (4.2)	0	23 (4.4)	0.99^*^
Former smoker	185 (33.7)	6 (28.6)	179 (33.9)	0.61^*^
Body-mass index (kg/m^2^)	28.7 [25.3–32.7]	27.6 [25.2–28.7]	28.8 [25.4–32.8]	0.023
*Comorbid conditions*				
Arterial hypertension	308 (56.1)	13 (61.9)	295 (55.9)	0.59
Diabetes	190 (34.6)	6 (28.6)	184 (34.8)	0.55
COPD	38 (6.9)	0	38 (7.2)	0.39^*^
Asthma	33 (6.0)	2 (9.5)	31 (5.9)	0.36^*^
Obstructive sleep apnea	63 (11.5)	0	63 (11.9)	0.15^*^
Interstitial lung disease	12 (2.2)	0	12 (2.3)	0.99^*^
*Medication before ICU admission*				
Corticosteroids^a^	350 (63.8)	13 (61.9)	337 (63.8)	0.86
Tocilizumab	20 (3.6)	0	20 (3.8)	0.99^*^
Time between symptoms onset and ICU admission (days)	9 [7–11]	9 [7–11]	9 [7–11]	0.71
Time between ward admission and ICU referral (days)	1 [0–3]	1 [0–3]	1 [0–3]	0.65
SAPSII score	31 [24–38]	31 [26–39]	31 [24–38]	0.57
*Biological parameters, day 1*				
WBC, G/L	8.3 [6.1–11.3]	7.2 [5–11.5]	8.3 [6.2–11.3]	0.37
Neutrophil, G/L	7.1 [4.9–9.6]	6.8 [4.4–9.9]	7.1 [5–9.6]	0.70
Lymphocyte, G/L	0.7 [0.5–1]	0.6[0.4–1.0]	0.7 [0.5–1]	0.29
Platelet, G/L	234 [172–300]	221 [172–285]	235 [172–300]	0.53
D-dimers, ng/mL	1380 [823–2530]	1425 [986–1886]	1368 [821–2570]	0.84
CRP, mg/L	120 [70–188]	121 [53–175]	120 [70–189]	0.77
LDH, IU/L	546 [415–738]	787 [682–984]	538 [415–731]	0.04
Creatinine, µmol/L	71 [57–92]	67 [59–79]	71 [56–93]	0.59
*Blood Gas, day 1*				
pH	7.46 [7.44–7.49]	7.48 [7.46–7.51]	7.46 [7.44–7.49]	0.11
PaO_2_/FIO_2_ (mmHg)	105 [72–153]	112 [96–158]	103 [72–153]	0.60
PaCO_2_ (mmHg)	34 [31–38]	32 [30–35]	34 [31–38]	0.04
*Baseline chest CT-scan*^b^				
Extent of lung damage > 50%	213 (46.1)	11 (64.7)	202 (45.4)	0.12
Emphysema	45 (9.7)	3 (17.6)	42 (9.4)	0.22
Pulmonary embolism	23 (5.6)	0	23 (5.8)	0.99^*^
Bronchiectasis/cyst	18 (3.9)	1 (5.9)	17 (3.8)	0.50^*^
*Immunomodulatory treatment during ICU stay*				
Corticosteroids	538 (98)	21 (100)	517 (97.9)	0.99^*^
Additional corticosteroid pulses	72 (13.1)	9 (42.9)	63 (11.9)	0.0006^*^
Time between ICU admission and corticosteroid pulses	9 [4–18]	12 [6–17]	9 [4–18]	0.66
Dose of corticosteroid pulses (mg/kg)^**c**^	2.7 [2–3.4]	3 [2.5–3.2]	2.7 [1.9–3.4]	0.58
Tocilizumab	16 (2.9)	0	16 (3)	0.99^*^
*Organ support during ICU stay*				
Awake prone positioning	139 (25.6)	12 (60)	127 (24.3)	0.0003
Noninvasive ventilation support				
HFNO	497 (90.5)	21 (100)	476 (90.2)	0.25^*^
Additional NIV to HFNO	161 (29.3)	11 (52.4)	150 (28.4)	0.02^*^
Invasive Mechanical Ventilation	249 (45.4)	11 (52.4)	238 (45.1)	0.51
Time between ICU admission and IMV, (days)	2 [1–5]	6 [5–13]	2 [1–4]	0.00002
Tracheostomy	44 (8)	2 (9.5)	42 (8)	0.68^*^
Vasopressor supports	177 (32.2)	11 (52.4)	166 (31.4)	0.04
ECMO	18 (3.3)	2 (9.5)	16 (3)	0.15^*^
Renal replacement therapy	62 (11.3)	3 (14.3)	59 (11.1)	0.72^*^
*Outcomes*				
Death in ICU	153 (27.9)	7 (33.3)	146 (27.7)	0.57
ICU length of stay (days)	10 [5–21]	18 [14–25]	9 [5–21]	0.008
Hospital length of stay (days)	19 [13–32]	27 [22–35]	18 [12–32]	0.01

Data are presented as median [first through third quartiles] or number (%). Continuous variables are compared using a Wilcoxon method; categorical variables are compared either using a *χ*^2^ test or Fisher’s exact test when followed by (^*^)

*IMV* Invasive mechanical ventilation; *WBC* White blood cell; *CRP* C-reactive protein; *LDH* lactate dehydrogenase; *CPK* Creatine phosphokinase; *ICU* Intensive care unit; *ECMO* Extracorporeal membrane oxygenation

^a^Initial corticosteroid therapy was either dexamethasone 6 mg or Hydrocortisone 200 mg per day

^b^Baseline CT scan was performed in 17 (80.9%) patients in SP group and 446 (84.6%) patients in control group (*P* = 0.65)

^c^Corticosteroid pulses dose in methylprednisolone equivalent

As compared with their counterparts, patients with SP had similar COVID-19 symptoms duration when admitted to the ICU, and displayed similar rates of preexisting chronic respiratory diseases or smoking status. Laboratory findings showed higher initial levels of lactate dehydrogenase, and blood gas showed lower PaCO_2_ upon ICU admission (Table 1). Baseline CT scan was performed after 0 day [0–1] following hospital admission; the disease extent was not different between cases and controls. The proportion of patients requiring IMV was similar between groups. However, the time to tracheal intubation was longer in patients with SP (6 days [5–13] vs. 2 days [1–4]; P = 0.00002), with a higher first-line use of non-invasive ventilation (NIV) (*n* = 11; 52% vs. *n* = 150; 28%; *P* = 0.02), and more awake prone positioning sessions (*n* = 12; 60% vs. *n* = 127; 24%; *P* = 0.0003). Patients with SP received more often vasopressors (*n* = 11; 52% vs. *n* = 166; 31%. *P* = 0.04) and had a longer ICU length of stay (18 [14–25] vs. 9 [5–21] days; *P* = 0.008), but ICU mortality rates did not differ between groups (*n* = 7; 33% vs. *n* = 146; 28%. *P* = 0.57) (Table 1).

**Fig. 1:**
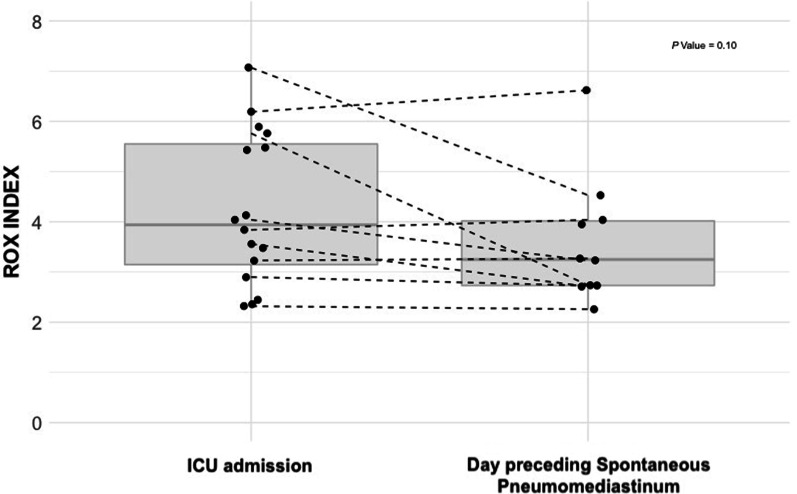
Evolution of the ROX index between ICU admission and the occurrence of SP. ROX index was at 3.94 [3.15–5.55] (missing data = 5) on ICU admission vs. 3.25 [2.73–4.02] (missing data = 11) the day preceding SP (*P* = 0.1)

When we focused specifically on the 21 patients who developed SP in order to investigate potential indirect signals of Patient-self inflicted lung injury (P-SILI), we noticed persisting signs of severe lung disease and respiratory failure: the extent of radiological lung damage and PaO2/FIO2 ratio did not significantly improve between the day of ICU admission and the day preceding SP. Additionally, the ROX index remained low with a trend toward a decrease along the time (3.94 [3.15–5.55] at ICU admission vs. 3.25 [2.73–4.02] the day preceding SP; *P* = 0.1) (Fig. 1).

## Discussion

In this multicenter series of critically-ill COVID-19 patients, the prevalence of SP without IMV was not uncommon, affecting 4% of patients admitted to the ICU with initial non-invasive respiratory support. Spontaneous pneumomediastinum has been previously described during SARS-CoV infection with an estimated prevalence of 12% of hospitalized patients [5, 6], and higher association with intubation and mortality rates [5]. In our series, SP was associated with higher need for vasopressors and a longer ICU length of stay, as reported in other series [7, 8].

The fact that SP occurs in the absence of IMV may suggest that the role of barotrauma related to IMV is not preponderant in its occurrence. One pathophysiological mechanism may potentially be carried out by P-SILI related to a prolonged respiratory failure [9], as underlined by a delayed use of IMV, a more marked initial respiratory alkalosis, and the evolution of ROX index between ICU admission and the day preceding SP. The more frequent use of NIV in patients who developed SP may also have promoted high transpulmonary pressures and unprotective tidal volumes. [10] The wider use of initial non-invasive respiratory supports has certainly allowed to decrease the dramatic first-line use of IMV in critically-ill COVID-19 patients, in the context of limited resources during the pandemic [11, 12]. However, it may have also led to inappropriate respiratory drive monitoring, and to the high risk of P-SILI and its complication. [13]

On the other hand, respiratory effort during severe COVID-19-related pneumonia managed by non-invasive respiratory support has been reported to be significantly lower than that usually observed during other etiologies of de novo acute hypoxemic respiratory failure [14–16]. The other intricate mechanism may be mediated by the severe and overwhelming lung inflammation, with significant cellular damages, as described in SARS-CoV infection [5, 17]. However, the baseline clinical characteristics, extent lung damage, and biological inflammatory markers (except higher LDH levels) were similar between patients with and without SP. This could imply that lung inflammation alone is not sufficient and the inspiratory effort may work as a “second hit” [9]. Indeed, both mechanisms could promote the development of SP by a “Macklin effect” through the destruction of the alveolar-capillary unit resulting in interstitial emphysema and air dissection along the pulmonary vasculature into the mediastinum [18]. The absence of isolated pneumothorax before intubation, as described during SARS CoV [5], supports the hypothesis of these two mechanisms affecting the pulmonary system in a diffuse way.

One of the main limitations of our study is related to its retrospective nature with missing data regarding respiratory rates during ICU stay and tidal volumes in patients receiving initial non-invasive respiratory supports. However, the large sample size of our population and the multicenter design are strengths. Whether COVID-19 SP is a contributor to adverse outcomes or a marker of disease severity remains unresolved. The presence of a SP should alert the ICU physicians of P-SILI in spontaneously breathing patients. In this context, clinicians may closely monitor signs of vigorous inspiratory efforts at risk of P-SILI. Indirect monitoring tools such as the ROX index during HFNO strategy or high tidal volume during NIV may help on the decision to apply of a more protective ventilation in a timely fashion.

## Data Availability

The datasets and materials used and/or analyzed during the current study are available from the corresponding author on reasonable request.

## Abbreviations

COVID-19: Coronavirus disease 2019
ICU: Intensive care unit
IMV: Invasive mechanical ventilation
NIV: Non-invasive ventilation
P-SILI: Patient-self inflicted lung injury
SP: Spontaneous pneumomediastinum
